# Effectiveness and tolerability of abacavir-lamivudine-nevirapine (ABC/3TC/NVP) in a multicentre cohort of HIV-infected, ARV-naïve patients

**DOI:** 10.7448/IAS.17.4.19773

**Published:** 2014-11-02

**Authors:** Daniel Podzamczer, Jhon Fredy Rojas, Isabel Neves, Elena Ferrer, Josep M Llibre, Manuel Leal, Miguel Gorgolas, Crusells M Jose, Josep M Gatell, Ricardo Correia Abreu, Jordi Curto, Pere Domingo, Barrufet M Pilar, Nerea Rozas

**Affiliations:** 1 Infectious Diseases, Hospital Universitari de Bellvitge, Barcelona, Spain; 2 Infectious Diseases, Hospital Clinic, Barcelona, Spain; 3 Infectious Diseases, Hospital Pedro Hispano – ULS Matosinhos, EPE, Porto, Portugal; 4 Infectious Diseases, Hospital Germans Trias i Pujol, Barcelona, Spain; 5 Infectious Diseases, Hospital Universitario Virgen del Rocio, Sevilla, Spain; 6 Infectious Diseases, Fundacio Jimenez Diaz, Madrid, Spain; 7 Infectious Diseases, Hospital Clinico Universitario Lozano Blesa, Zaragoza, Spain; 8 Infectious Diseases, Hospital de la Santa Creu i Sant Pau, Barcelona, Spain; 9 Infectious Diseases, Hospital de Mataro, Mataro, Spain

## Abstract

**Purpose:**

Very scarce information has been published to date with the combination of ABC/3TC/NVP but it is currently being used in clinical practice in Spain and Portugal. Our aim was to present the clinical experience with this regimen in a cohort of adult HIV-infected antiretroviral (ARV)-naïve patients.

**Methods:**

Retrospective, multicentre, cohort study. Consecutive adult HIV-infected ARV-naïve HLA-B*5701-negative patients, who started ABC/3TC/NVP between 2005-2013, with at least one follow-up visit, were included. Demographic, clinical and laboratory variables were assessed at baseline, month 1, and every three–four months thereafter. The primary end point was HIV-1 viral load (VL)<40 c/mL at 48 weeks. Data were analyzed by intent-to-treat (ITT) (switch=failure, and missing=failure) and on treatment (OT) analyses.

**Results:**

78 patients were included. Median follow up was 26 (0.1-84) months. 86% were male, median age 41 (23-69) years, 9% had AIDS, 8% were HCV+, baseline CD4 was 275 (10-724) cells/µL and median VL 4.58 (3.02-6.92) log. After 48 weeks, VL was<40 c/mL in 89.8% (OT), 79.7% (M=F) and 65.4% (S=F) and at 96 weeks in 88.5%, 78.9% and 61.6%, respectively. CD4 increased +246 (p<0.001) and +292 (p<0.001) cells/uL after 48 and 96 weeks, respectively. One or more drugs of the regimen were discontinued in 33 (42.3%) patients. In 15 (19.2%) patients (13 NVP, 2 ABC/3TC) therapy was stopped due to toxicity after a median of one month (in only two cases after six months of follow up): 80% of them had rash/liver toxicity. Six (7.7%) patients discontinued ART due to virologic failure, five (6.4%) because of other reasons and seven (9%) were lost to follow-up. ALT but not AST significantly increased (+0.07 ukat/L at 96 weeks, p=0.033). A significant increase of 25%, 26% and 42% in total cholesterol, LDLc and HDLc, respectively, and a significant decrease in TC/HDL ratio (6%, p=0.008) was observed after 96 weeks.

**Conclusions:**

Despite a considerable proportion of patients had to stop therapy due to toxicity (most associated with NVP), those initially tolerating this regimen presented a high virologic and immunologic response after 96 weeks, as well as a favourable lipid profile. ABC/3TC/NVP may be a suitable alternative first regimen, mainly in countries with economic constraints.

